# An Immunological Stairway to Severe Tissue Complication Assembly in *Bothrops atrox* Snakebites

**DOI:** 10.3389/fimmu.2019.01882

**Published:** 2019-08-13

**Authors:** Hiochelson Najibe Santos Ibiapina, Allyson Guimarães Costa, Jacqueline Almeida Gonçalves Sachett, Iran Mendonça Silva, Andréa Monteiro Tarragô, Juliana Costa Ferreira Neves, Marllon Wendell Athaydes Kerr, Monique Freire Santana, Olindo Assis Martins-Filho, Marcus Vinícius Guimarães Lacerda, Luiz Carlos Lima Ferreira, Adriana Malheiro, Wuelton Marcelo Monteiro

**Affiliations:** ^1^Programa de Pós-Graduação em Medicina Tropical, Universidade do Estado do Amazonas (UEA), Manaus, Brazil; ^2^Instituto de Pesquisa Clínica Carlos Borborema, Fundação de Medicina Tropical Doutor Heitor Vieira Dourado (FMT-HVD), Manaus, Brazil; ^3^Programa de Pós-Graduação em Imunologia Básica e Aplicada, Instituto de Ciências Biológicas, Universidade Federal do Amazonas (UFAM), Manaus, Brazil; ^4^Diretoria de Ensino e Pesquisa, Fundação Hospitalar de Hematologia e Hemoterapia do Amazonas (HEMOAM), Manaus, Brazil; ^5^Programa de Pós-Graduação em Ciências Aplicadas à Hematologia, Universidade do Estado do Amazonas (UEA), Manaus, Brazil; ^6^Grupo Integrado de Pesquisas em Biomarcadores de Diagnóstico e Monitoração, Centro de Pesquisas René Rachou, Fundação Oswaldo Cruz (FIOCRUZ), Belo Horizonte, Brazil; ^7^Instituto de Pesquisas Leônidas & Maria Deane, FIOCRUZ-Amazônia, Manaus, Brazil

**Keywords:** blister, immune response, *B. atrox*, Brazilian amazon, immunological molecules

## Abstract

Snakebites are a serious public health problem and, in the Amazon, the *Bothrops atrox* snake is the most frequent cause of envenomation. *B. atrox* venom (BaV) causes pathophysiological changes with intense, local inflammatory processes, such as severe tissue complication (STC). However, mechanisms associated with the inflammatory process in humans are still poorly understood. Thus, in this study, we sought to describe the profile of local and systemic immunological soluble molecules in *Bothrops* envenomation patients treated at a specialist tertiary healthcare unit in the Brazilian Amazon. An analytical and prospective study was performed with patients who had snakebites with different clinical outcomes (STC and Mild Tissue Complication—MTC) using venous blood and blister exudate in order to measure immunological soluble molecules present in the response process. Twenty STC patients and 20 MTC patients were eligible for the study. In addition, 20 healthy donors (HD) who had never been bitten by a snake were used as controls. The biomarkers CXCL-8, CCL-5, CXCL-9, CCL-2 and CXCL-10; C3a, C4a, and C5a; IL-1, IL-2, IL-4, IL-5, IL-6, IL-10, TNF, IFN-γ and IL-17A were quantified using flow cytometry and ELISA. The circulating response profile differs between the studied groups, with MTC patients presenting a mixed profile and STC patients presenting a more polarized profile for Th1 response. In addition, individuals who develop STC have a more intense local immune response, because the tissue response differs from the circulating immunological soluble molecules and presents Th1/Th2/Th17 response polarization. Furthermore, these results suggest that CCL-2 and CXCL-10 are biomarkers for STC and the response profile they assume against *Bothrops* snakebite should reflect in the clinical practice for the patient.

## Introduction

Snakebites frequently occur around the world, causing great morbidity and mortality problems (1–5). Men of productive age (18–60 years), inhabitants of poor regions involved in agricultural activities are the most affected group (4, 6–11). The number of annually reported cases in the world ranges from 1.8 to 2.7 million envenomations, and these cause 81,000–138,000 deaths, thus representing a serious public health problem (2, 5). In Brazil, *Bothrops* snakes cause approximately 90.5% of reported envenomations (12, 13).

*Bothrops* venom is mainly constituted of phospholipase A2 (PLA2), metalloproteases (MPs) and serine proteases (SPs) that cause classic symptoms such as hemorrhage, clotting and acute inflammation due to its phospholytic and proteolytic action (14–16). The MPs are the main components of the *B. atrox* venom (BaV), and are accountable for causing damage to the basement membrane of the capillaries, and degrading type IV collagen, extracellular matrix components and proteoglycans (17). These may lead to extravasations and blister formation close to the site of bite (18–20).

The damage to the structure of the basement membrane of the epidermis leads to blister formation, which can be an aggravating factor and can facilitate the entry of microorganisms and thus lead to secondary infection. This was observed in 6.6% of the envenomations recorded in clinical-epidemiological cases in the Amazon, where a blister was present in 83% of secondary infections that were reported (21).

Experimental models show that the inflammatory process caused by BaV leads to an increase in the total number of leukocytes, mainly polymorphonuclear and/or mononuclear cells (22). Similarly, the presence of systemic and local immune changes in response to BaV showed an increase of local cytokines IL-6, IL-12p70, and IL-10 (23–25). In humans, a similar effect occurs with an increase in circulating IL-6, IL-10, CXCL-8 (IL-8), and MIP-1 (CCL-3/CCL-4), as this process is related to induction of immune response, fever, cell migration and profile regulation (26). Furthermore, specific components of BaV such as MP *batroxase* also proved capable of directly cleaving components of the complement system (C3, C4, and C5), leading to uncontrolled production of anaphylatoxins, that play an important role in the progression of signs and symptoms after envenomation (27).

Thus, the characterization of circulating and blister exudate chemokines, anaphylatoxins, and cytokines involved in the inflammatory process in victims of *B. atrox* snakebite becomes important, since such analyses might be a possible predictive factor for the clinical evolution of the patients, which would consequently influence their hospitalization time. Data presented in this study suggest that the CCL-2 and CXCL-10, molecules may be potential biomarkers and thus predictors of severe tissue complications in *Bothrops* envenomation's.

## Materials and Methods

### Study Area

The study was carried out at *Fundação de Medicina Tropical Dr Heitor Vieira Dourado* (FMT-HVD) and *Fundação Hospitalar de Hematologia e Hemoterapia do Amazonas* (HEMOAM) between June 2014 and June 2017.

### Patients and Sampling

**Figure 1:**
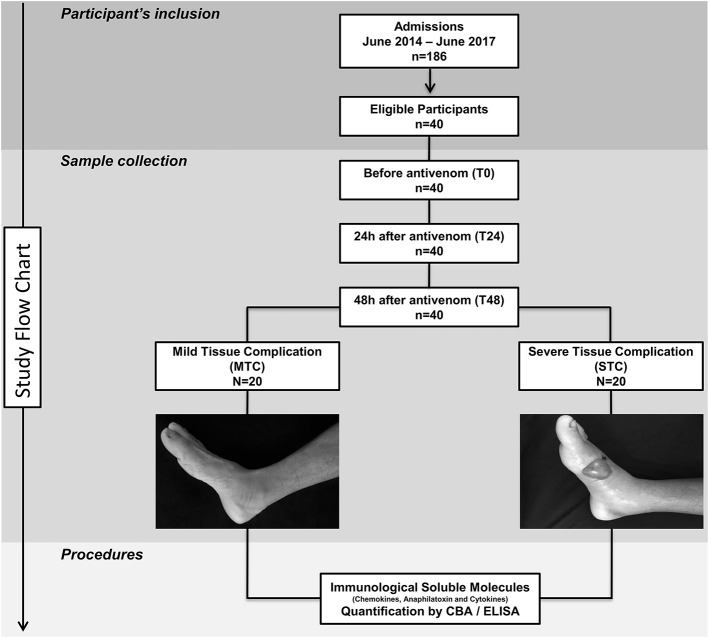
Flowchart of study. Forty patients were eligible and followed up until discharge. These patients were divided into two groups: Mild Tissue Complication (MTC) and Severe Tissue Complication (STC), according to their clinical outcome. STC patients developed a blister and had transudate collected 48 h after antivenom administration.

The study population consisted of 186 individuals who had been clinically diagnosed with a snakebite (acute inflammatory action and hemorrhagic or coagulant signs) caused by a *Bothrops* snake and who sought medical assistance at FMT-HVD. *Bothrops* identification was performed by a zoologist from the research group. We did not include pregnant women, indigenous people or individuals who reported a history of chronic inflammatory disease, autoimmune diseases or immunodeficiency. Patients were classified using to the Brazilian Health Ministry guidelines in mild (local pain and/or swelling and bruising), moderate (local manifestations without necrosis and minor systemic signs, such coagulopathy and bleeding) and severe cases (severe bleeding, hypotension, shock and/or acute renal failure) (13). The included patients (40 samples) were divided into two subgroups: Mild Local Complication (MTC), classified as mild envenomation and without presenting severe manifestations in the first 48 h after receiving antivenom; Severe Local Complication (STC), with moderate or severe envenomation and local manifestations such as purpura, ecchymosis, severe edema, blister formation and necrosis at the bite site within 48 h after receiving antivenom (Figure 1). In addition, 20 healthy patients of both genders, without a history of having been bitten by a snake, composed the control group at HEMOAM.

### Ethical Issues

This study was approved by the Ethics Review Board at FMT-HVD (CEP/FMT-HVD process #492.892, CAAE #19380913.6.00005016). Participants read and signed the written informed consent form before enrollment, according to the Declaration of Helsinki and Resolution 466/12 of the National Health Council for research involving human subjects (Supplementary Material). All patients were treated according to Brazilian Ministry Health recommendations (13).

### Biological Sample Collection and Data

Approximately 4 mL of peripheral blood was collected before antivenom administration (T0), 24 h (T24) and 48 h after antivenom administration (T48) by venous puncture in tubes containing EDTA (BD Vacutainer® EDTA K2). Blister exudate samples from STC patients was collected at T48 by aspiration, volume varied from 200 to 300 μL (Figure 1). The samples were stored in a freezer at −80°C for detection of the presence of envenomation and subsequent measurement of chemokines, anaphylatoxins and cytokines. Furthermore, clinical and epidemiological data of the patients were obtained using a standardized form.

### *Bothrops* Envenomation Diagnosis by ELISA

The diagnosis of *Bothrops* envenomation was confirmed by ELISA (Enzyme-Linked Immunosorbent Assay) qualitative specific-genus at the Instituto Butantan (IBu) as described previously (28).

### Circulating and Blister Exudate Immunological Soluble Molecules Level Quantification by CBA and ELISA

The circulating and blister exudate soluble molecules (CXCL-8, CCL-5, CXCL-9, CCL-2, CXCL-10, C3a, C4a, C5a,IL-2, IL-4,IL-6, IL-10, TNF, IFN-γ, and IL-17A) were quantified using CBA (Cytometric Bead Array). BD™ Human Chemokine kit (Code N° 552990, BD® Biosciences, San Diego, CA, USA), BD™ Human Anaphylatoxins Kit (Code 561418, BD® Biosciences, San Diego, CA, USA) and BD™ Human Th1, Th2, Th17 Cytokine Kit (Code 560484, BD® Biosciences, San Diego, CA, USA) were used following the manufacturer's technical guidelines and protocols. A FACS Canto II flow cytometer (BD® Biosciences, San Jose, CA, USA) at HEMOAM was used for sample acquisition. FCAP-Array software v3 (Soft Flow Inc., USA) was used to calculate the cytokine levels (MFI). The ELISA was performed for the quantification of IL-1β and IL-5 cytokines present in plasma and transudate. The Human BD™ OptEIA® Set II Kit was used for IL-1β cytokines (Code 557953, BD® Biosciences Pharmingen, San Diego, CA, USA) and IL-5 (Code 555202, BD® Biosciences Pharmingen, San Diego, CA, USA) following the manufacturer's technical guidelines and protocols. Dosages of soluble molecules were carried out at three different time intervals: T0, T24, and T48; and the collection of blister exudate samples at T48. Furthermore, plasma and blister exudate samples were analyzed without performing dilutions.

### Data Analysis

Statistical analyses were performed with the software GraphPad Prism (v5.0), Stata (v13.0), and R (v3.5.2). Data normality tests were done using the Shapiro-Wilk test for each variable and showed a non-parametric distribution. The Principal Component Analysis (PCA), which consists of a multivariate type of analysis, was based on the Z value generated by the score based on the mean and standard deviation of the components' variables. The PCA included the all soluble molecule variables of the patients before treatment. The coefficient correlation factor is considered significant when *p* < 0.05. The comparison of values between two data groups were performed using the Mann–Whitney test, whereas for comparison of the variables with three or more groups, the data analysis was performed using the Kruskal–Wallis test, followed by Dunn's post-test for multiple comparisons between groups. The general signature of high immunological soluble molecules produced by *Bothrops* envenomation was assembled as reported previously (29). In short, the global median value for each of the chemokines, anaphylatoxins, and cytokines was calculated using data from all of the patients (HD, MTC, and STC group). The global median for each immunological soluble molecule was employed as a cut-off point expressed in MFI (CXCL-8 = 627.77; CCL-5 = 187303.73; CXCL-9 = 2380.24; CCL-2 = 1229.80; CXCL-10 = 3071,64; C3a = 1164,74; C4a = 5469,04; C5a = 24834,10; IL-6 = 410.41; TNF = 104.38; IL-1β = 0.02; IL-10 = 122,98; IL-2 = 154,62; IFN-γ = 92,20; IL-4 = 165,38; IL-5 = 0,05; and IL-17A = 115,24) in order to discriminate each individual as being a “Low” or “High” producer of the chemokines, anaphylatoxins and cytokines summarized in the grayscale diagrams. Afterwards, the “immunological soluble molecule signature” for the HD group was then assembled and taken as the “reference curve” (-□-) to highlight the changes in the chemokines, anaphylatoxins and cytokines profile of MTC and STC. The relevant differences considered when the values were above the 50th percentile of the study group was confined to the “higher producers.” The Venn Diagram was created using the website [http://bioinformatics.psb.ugent.be/webtools/Venn/]. A Spearman correlation test was performed and then the networks were built with Cytoscape 3.0.3 software (Cytoscape Consortium San Diego, CA, USA), following the recommendations and instructions in the software. The positive and negative correlations are considered significant when *p* < 0.05. The correlation index (r) was used to categorize the correlation strength as being weak (*r* ≤ 0.35), moderate (*r* ≥ 0.36 to *r* ≤ 0.67), or strong (*r* ≥ 0.68), as previously described (30, 31). The levels of statistical significance defined in both cases were *p* < 0.05.

## Results

### Clinical and Epidemiological Baseline of the Patients

Table 1 summarizes the clinical and epidemiological characteristics of the patients. Males were more frequently bitten, with similar mean age for both groups. Most snakebites occurred in rural areas, with only 12% of the patients reporting previous snakebites. The main anatomical site of the bite was the lower limbs. The MTC group had a mild classification, while in the STC group the bites were classified as moderate and severe. The time between the bite occurring and the patient at the medical center was no different on average between snakebite groups.

**Table 1:** Description of the socio-demographic data of HD, MTC, and STC groups.

**Variables**	**HD** ***n* = 20**	**MTC** ***n* = 20**	**STC** ***n* = 20**	***p*-value**
**Gender**, ***n*** **(%)**				
Male	13 (65)	14 (70)	18 (90)	0.154
Female	7 (35)	6 (30)	2 (10)	
Age (years, median [IQR])	27 [22–36]	31 [24–46]	38 [22–55]	0.372
**Previous snakebite**, ***n*** **(%)**				
Yes	–	2 (10)	3 (15)	0.063
No	–	18 (90)	17 (85)	
**Occurrence zone**, ***n*** **(%)**				
Rural	–	20 (100)	17 (85)	0.071
Urban	–	–	3 (15)	
**Classification**, ***n*** **(%)**				
Mild	–	20 (100)	–	<0.0001
Moderate	–	–	15 (75)	
Severe	–	–	5 (25)	
**Anatomical site of the bite**, ***n*** **(%)**				
Hand	–	2 (10)	1 (5)	0.596
Leg	–	2 (10)	4 (20)	
Foot	–	16 (80)	15 (75)	
Time sting/antivenom (hours, median [IQR])	–	2 [1–3]	2 [2–5]	0.570

### Clustering of Study Groups by Principal Component Analysis (PCA)

**Figure 2:**
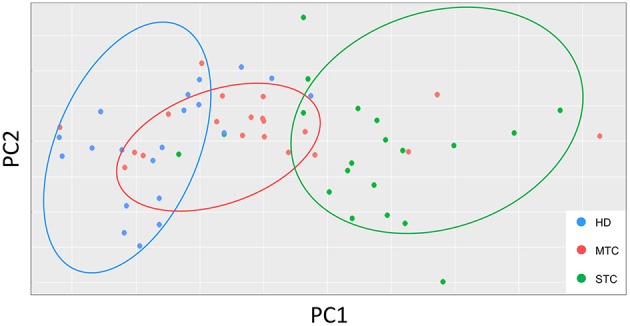
PCA graphs show the distribution of each patient according to their intragroup similarity and between groups. PCA graphs consist of a multivariate type analysis based on the Z value and included the all soluble molecule variables of the patients before treatment. The coefficient correlation factor is considered significant when *p* < 0.05.

To identify the dynamics of production, performance of immunological soluble molecules and describe a possible STC prognostic biomarker, the PCA analysis was performed based on the circulating levels of each molecule. According to the dispersion of the points, it is possible to observe clusters of each of the groups, which means that the classification of the participants in the research, even at random, reveals that the immunological parameters reflect the clinical characteristics. Distribution of each point on the PCA chart represents one patient, so that, after reducing the variables, the two-dimensional chart demonstrates that there is a similarity between patients within each group (Figure 2). The PC1 and PC2 represents CCL-2 and CXCL-10 molecules.

### STC *B. atrox* Snakebite Patients Presented Different Serum Levels of Immunological Soluble Molecules When Compared to Those With MTC and Those of the HD Groups

**Figure 3:**
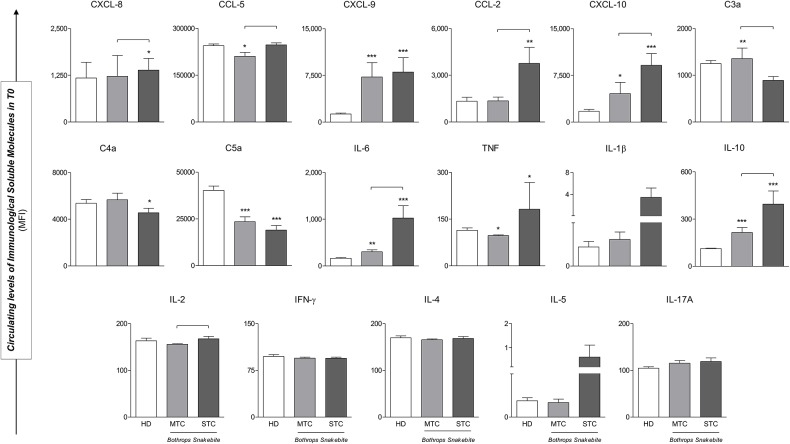
STC *Bothrops* snakebite patients presented different circulating levels of immunological soluble molecules when compared to those with MTC and those of the HD groups in baseline (T0). Significant difference from the HD group *p < 0.05; **p < 0.01; ***p < 0.0001; Statistical difference between MTC and STC groups, *p* < 0.05 was considered significant (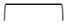). Immunological Soluble Molecules Level Quantification by CBA and ELISA. Data are expressed as mean ± standard deviation in Mean Fluorescence Intensity (MFI) for Immunological Soluble Molecules. Statistical analyses were performed by the Kruskal-Wallis test, followed by Dunn's test in order to compare pairs.

Levels of CXCL-9, CXCL-10, IL-6, and IL-10 immunological soluble molecules were higher in snakebite patients (MTC and STC) when compared to the HD group in baseline (T0). The levels of CXCL-8, CCL-2, and TNF are significantly elevated only in the STC group. Regarding the anaphylatoxins, only C5a is decreased in STC and MTC groups. In the comparison between patient groups, higher circulating concentrations of chemokines (CXCL-8, CCL-5, CXCL-10 and CCL-2) and cytokines (IL-6, IL-10 and IL-2) were found in STC group when compared to MTC. Furthermore, anaphylatoxins C3a was found to have lower concentrations in the STC group (Figure 3). Although, IL-1β and IL-5 concentrations were higher in the STC group, there was no significant difference (*p* = 0.401 and *p* = 0.205 respectively). Median and interquatil range (IQR) of immunological soluble molecules were described in Supplementary Table 1.

### Signature of Immunological Soluble Molecules Demonstrated in STC Patients

**Figure 4:**
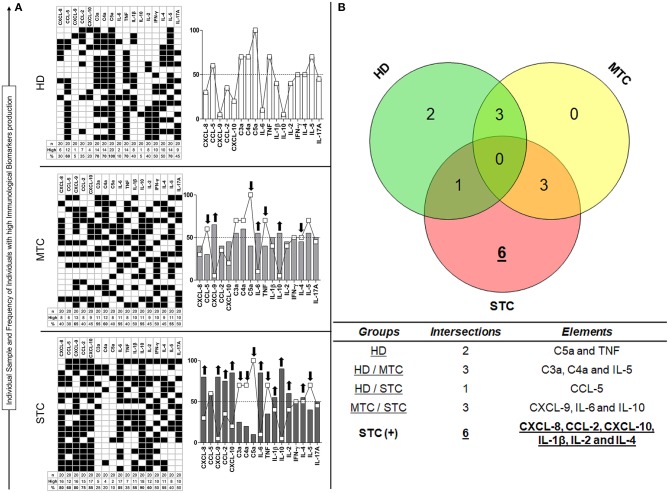
Unconventional analysis for signature of biomarkers demonstrated in STC patients. This is shown on the basis of the total percentage of each group, in which molecules present higher production (↑) in each patient (■), considering the clinical outcome **(A)**. In the Venn diagram, it is possible to identify which elements demonstrate themselves to be potential biomarkers by being present in high concentrations in the STC group only **(B)**. Immunological Soluble Molecules Level Quantification by CBA and ELISA. The global median for each immunological soluble molecule was employed as a cut-off point expressed in MFI to discriminate each individual as being a “Low” or “High” producer of the chemokines, anaphylatoxins, and cytokines summarized in the grayscale diagrams.

Figure 4 shows the immunological biomarker identification of STC patients with *B. atrox* snakebites. The proportion of individuals with high levels of soluble molecules in MTC and STC groups characterize a typical immunological storm. Data analysis indicated a significant increase in the proportion of patients with high soluble molecule levels in both snakebite groups. MTC group had a profile of individuals with high production of the molecules CXCL-9, IL-6, and IL-10, whereas the STC group presented a profile of high producers of the molecules CXCL-8, CXCL-9, CCL-2, CXCL-10, IL-6, IL-1β, IL-10, and IL-2, an inverse profile to that observed in the HD group (Figure 4A). Figure 4B summarizes the identification of immunological biomarkers with a Venn diagram, giving an objective view of which molecules have a similar (or unique) pattern in each group. The analysis of the number of intersections of each element demonstrated that CXCL-8, CCL-2, CXCL-10, IL-1β, IL-2, and IL-4 are potential biomarkers for STC group (Figure 4B).

### Follow-Up of the Production of Immunological Soluble Molecules in *B. atrox* Snakebite Patients

**Figure 5:**
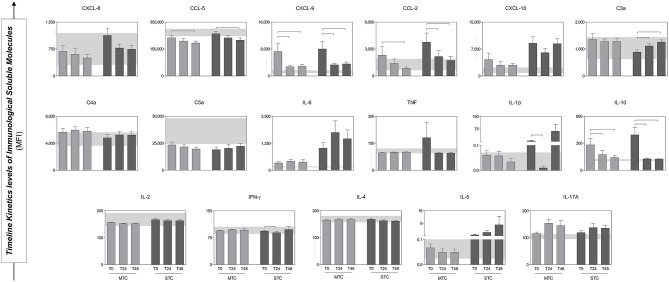
Serum concentrations of Immunological Soluble Molecules of patients (MTC and STC) during the follow up. At the bottom, the interquartile range (25–75) of the serum concentrations in the HD group is shown as the baseline parameter. Statistical difference between MTC and STC groups, *p* < 0.05 was considering significant (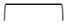). Immunological Soluble Molecules Level Quantification by CBA and ELISA. Data is expressed as mean ± standard deviation in MFI for Immunological Soluble Molecules. Statistical analyses were performed by the Kruskal–Wallis test, followed by Dunn's test in order to compare pairs.

The analysis of the production dynamics of immunological soluble molecules was performed at T0, T24, and T48 (Figure 5). The comparison between T0, T24, and T48 in each group more reliably describes the chemokine, anaphylatoxins and cytokine profiles and how they behave during follow-up. In general, the chemokines and cytokines levels were higher at T0 in both groups and showed a decrease in circulating concentration over time (e.g., CXCL-8 and IFN-γ), except for the molecules C4a, C5a, IL-6, IL-5, and IL-17A that demonstrate an increase during hospitalization, without significant difference. However, CCL-5, CXCL-9, CCL-2, and IL-10 presented significant decrease in circulating concentration, while there was significant increase of C3a.

### STC *B. atrox* Snakebite Patients Presented Different Blister Exudate Concentration of Immunological Soluble Molecules When Compared to Circulating Serum

**Figure 6:**
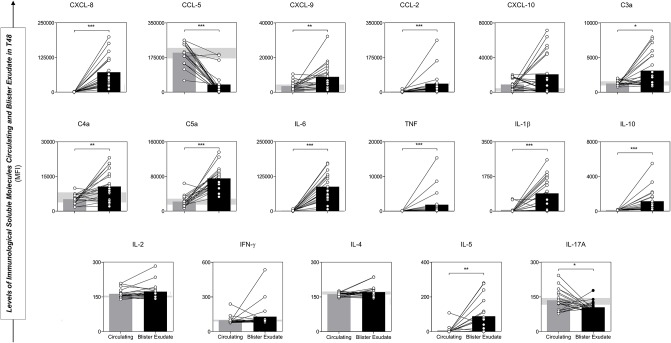
Comparative analysis of Immunological Soluble Molecules circulating and blister exudate paired samples from STC patients illustrated the production profile and concentration in the different environments. Values of *p* < 0.05. *p < 0.05; **p < 0.01; ***p < 0.0001 were considered significant. Immunological Soluble Molecules Level Quantification by CBA and ELISA. Data are expressed as mean ± standard deviation in MFI for Immunological Soluble Molecules. Statistical analyses were performed using the Mann-Whitney test.

Analysis performed between circulating serum and blister exudate concentrations of chemokines, anaphylatoxins and cytokines demonstrate a different pattern according to the site studied, which depicts the uniqueness of the immune response within the same (STC) group (Figure 6). The concentration of chemokines (CXCL-8, CXCL-9, and CCL-2), anaphylatoxins (C3a, C4a, and C5a), inflammatory and regulatory cytokines (IL-6, TNF, and IL-10) were statistically higher in the blister, with a tendency to mixed pattern. However, signaling with increased inflammatory power at the circulating level.

### *B. atrox* Snakebite Patients Demonstrated Complex Biomarker Networks With Rich Interactions Between Circulating Serum and Blister Exudate Markers

**Figure 7:**
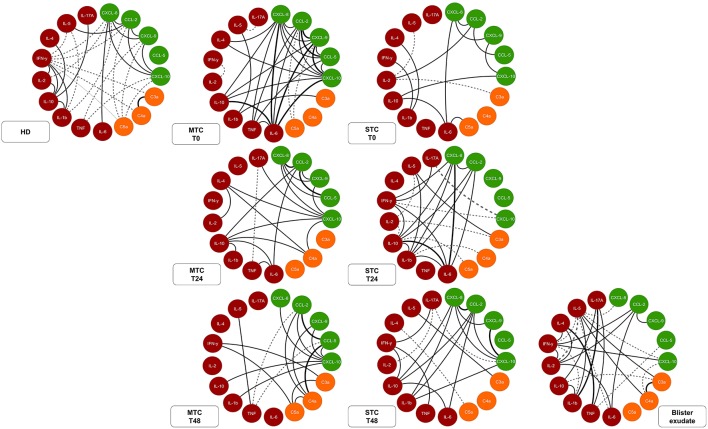
Network of soluble molecules shows interactions occurring throughout the clinical evolution of MTC and STC patients, interaction of the molecules in the control T0 (HD) and interactions at the site (Tissue). Each group of colored nodes is used to identify chemokines (green), anaphylatoxins (orange), and cytokines (red). Dashed lines indicate negative correlation and continuous lines in black, positive correlation, while thickness shows correlation strength. The correlation index (r) was used to categorize the correlation strength as weak (*r* ≤ 0.35), moderate (*r* ≥ 0.36 to *r* ≤ 0.67), or strong (*r* ≥ 0.68). Immunological Soluble Molecules Level Quantification by CBA and ELISA.

Data analysis demonstrated that MTC and STC patients have an immunological soluble molecule network different from that exhibited by the HD group (Figure 7). In T0, correlations in the MTC, demonstrate an acute inflammatory response occurring with strong and moderate correlations between chemokines and inflammatory cytokines, with regulation promoted by IL-10. Decreases in the correlations between the inflammatory cytokines and the beginning of a more regulatory process with increased correlations were observed in MTC patients at T24 and T48. Furthermore, these patients presented a predominance of a massive chemotactic process with many moderate and strong correlations between chemokines and anaphylatoxins, as well as a more polarized profile for Th1 response at the end of follow-up. STC patients demonstrated a poor interaction between chemokines, anaphylatoxins, and cytokines at T0. Contrary to that observed in the MTC group, in T24 and T48, this group a more evident inflammatory response began, with increasing interaction of chemokines and inflammatory cytokines, besides Th1 and Th17 profile interaction. Furthermore, after the antivenom administration, these patients presented increased correlations of the chemokines with the immunological soluble molecules during clinical attendance. In the blister exudate, a different profile of the circulating serum was observed, with a wide range of negative correlations with mixed profile molecules that were present. Moreover, only chemokine CCL-2 had a correlation with inflammatory cytokines as well as anaphylatoxins, which characterizes an acute inflammatory process, with the presence of markers in the Th1, Th2, and Th17 profiles.

## Discussion

Blister formation can occur due to damage to the basement membrane structure, especially in laminins, type IV collagens, and integrins, as well as other molecules. Metalloprotease of *B. asper* venom (BaP-1) was capable of causing local tissue damage, inflammation, and destruction of the basement membrane (32). Furthermore, it was observed that this metalloprotease was able to induce the formation of blisters, and may lead to dermo-epidermal junction detachment resulting in inflammatory infiltration with predominance of macrophages (33).

Studies describe the action of some cytokines in envenomation's caused by animals such as snakes and scorpions, thus emphasizing the acute inflammatory action caused by the components of the venom (34–36). The initial process of acute inflammatory response caused by the venom includes the involvement of endogenous mediators (LTB4 and PGE2) that lead to cellular activating of macrophages and regulate the production of many cytokines (37). Pattern recognition receptors (PRRs) such as TLRs are primordial in this initial response process. Moreira et al. described the importance of TLR-2, TLR-4, and the CD14 in the inflammatory process of the venom of *B. atrox* (23). Furthermore, PAMPs and DAMPs may also be molecular patterns associated with venom (VAMPs), which could partially explain why such a strong innate response to venom, mediated by PRRs, could lead to the production of inflammatory molecules (38).

Macrophages may have a significant role in recognition of these VAMPs and the consequent production of cytokines and chemokines, both directly and indirectly, mainly TNF-α and IL-6, which play an important role in the inflammatory process (39). These findings could justify the elevated levels of TNF and IL-6 cytokines found in both the circulating and blister exudate in STC patients, probably due to the number of resident macrophages in the skin, which are specialized phagocytes and have functional TLRs in their surface. In addition, these cytokines were significantly elevated in the STC patients when compared to the HD and MTC groups.

Higher levels of IL-1β, IL-6, and TNF-α were associated with the severity of the snakebite, producing moderate/severe characteristics according to higher values of these cytokines, when compared to mild envenomation's (34). This data corroborates our findings, since the STC patients presented increased values of these cytokines when compared to the MTC group. It is noteworthy that in envenomation's caused by *Loxosceles intermedia*, high values of TNF-α demonstrated a correlation with mortality (40).

*Bothrops* envenomation's have marked clinical aspects represented by the presence of pain, edema, hemorrhagic/coagulant action, and presence of inflammatory infiltration due to the acute inflammatory action of the venom (20, 41, 42). The clinical aspects in these envenomation's are very similar, irrespective of the species, and present the following as main, local signs and symptoms: edema, myonecrosis, vascular damage, ischemia, hemorrhage and inflammation. This pattern of clinical signs is replicated in *B. jararaca* (43), *B. erythromelas* (44), *B. alternates* (44), *B. pirajai* (45), *B. asper* (46), and *B. atrox* (23).

In animal experiments, it has been shown that the inflammatory infiltration in the first 24 h after inoculation of the venom is represented mainly by neutrophils, and then by monocytes/macrophages (22). Thus, these cells would be mainly responsible for the process of blister exudate and circulating serum response (23, 46). In addition, in *B. atrox* envenomation's in BALB/c mice, the presence of neutrophils may influence levels of IL-1β (decrease), IL-6, MIP-1 (CXCL-1), and MIP-2 (CXCL-2) (47). This cell type is described as the first to reach the site of the damage due to elevated levels of CXCL-8, which is one of the first chemotaxis molecules produced in the event of injury (48).

Knockout mice for MyD88 had lower levels of CCL-2 and cytokines of the Th1 and Th17 profile, which reinforces the importance of the innate response and the role of PRRs in the process of immune response to *B. atrox* venom (24). Our results demonstrate that high values of CCL-2 were found only in the STC group at systemic T0 and at the bite location 48 h after receiving antivenom. IL-6 may be one molecule involved in the prognosis of more severe, local conditions (26). This cytokine showed significantly elevated levels in both snakebite groups when compared to the HD group in our study. However, CCL-2 only showed significantly elevated levels in the STC group, which highlights it as a potential prognostic molecule and involved with severe tissue complication.

Some studies describe an increase in CCL-5 and CXCL-8 concentrations in *Bothrops* snakebites (26, 49). Our findings demonstrate an increase in the circulating levels of these chemokines in the STC group, which could be influencing the acute inflammatory response, since these patients present a more marked local inflammation up to 48 h after the envenomation. Furthermore, a type C lectin, called galatrox, present in the venom of *B. atrox* can be recognized by macrophages by means of TLR-4, leading to the production of IL-6 and TNF-α, which reinforces the idea of the existence of VAMPs (50). Also, the presence of galatrox increases the activating of the pathways of the complement system (lectin pathway) leading to an increase in the neutrophil chemotaxis process due to the release of anaphylatoxins.

We observed a decrease in the levels of anaphylatoxins in the STC group, with statistically significant decreases in C5a levels of STC and MTC patients when compared to the HD group, besides a low concentration of C3a only in the STC groups when compared to the MTC. Menaldo et al. showed that the total venom of *B. atrox* and an MP called batroxase can have a direct action on molecules of the complement system (C3, C4, C5, and Factor B), directly cleaving them. Thus, we would have the release of anaphylatoxins regardless of the pathway of action (27). Moreover, The class P-I MP of *B. pirajai* would have the ability to directly cleave core components of the complement system (C3, C4, and C5) (51, 52).

This decrease in the levels of anaphylatoxins demonstrated by our results may be a consequence of rapid consumption of these molecules by neutrophils and monocytes/macrophages, which would be in agreement with the rapid activation and performance of these cells, systemically and locally, as described in other studies (22, 23, 46). In addition to the chemokines, anaphylatoxins also participate in the cellular recruitment process due to the direct action of both the total venom and the batroxase and galatrox portions in the central components of the complement system (C3, C4, C5, and Factor B).

In experimental models, there is the suggestion that C3a can mediate the migration and adhesion of leukocytes to the site of the envenoming, C5a and / or CXCL-8, which leads to the increase of expression of molecules involved in diapedesis (P-selectin and E-selectin) and consequent cell adhesion due to elevated levels of TNF-α (53). Our results demonstrate that, at T48, we found high levels of the chemokines, cytokines CXCL-10, TNF, IL-6, IL-1β, and INF-γ, therefore, due to this, we suggest that there is a polarization of macrophages to M1 profile, since the patient still has a strong, local, inflammatory site even at 48 h after the incident. CXCL-10, TNF, IL-6, IL-1β, and INF-γ are linked with macrophage polarization to the M1 profile (54, 55), acting together with complement molecules C3a and C5a, that are also linked to the polarization of this profile in macrophages (56).

Our data demonstrate that there is an increase in TNF and anaphylatoxins in the bite location, thus reinforcing that damage may have some influence on the soluble molecule tissue. Thus, the direct action of the venom on complement molecules, cleaving them, and acting as “TNF convertase” could be an aggravating factor for local damage (57). The blistering formed by the action of components of envenomation could function as a venom reservoir, thereby causing the local inflammatory process in STC patients to be more intense. We observed that there is a synergism of these molecules with the cellular profile, and that, to support polarization Th1 in STC group (T0), the presence of molecules, such as IL-12 and IFN- in the bite location is necessary.

The signature graph demonstrates that there is a positive correlation between molecules of different response profiles (Th1, Th2, and Th17 or mixed patterns), thus suggesting that the local response has a plurality in the response profile. However, although Th1, Th2, and Th17 are present, there is a predominance of Th1 profile molecules. These findings suggest that the circulating response profile has a polarization to the Th1 profile, as presented by Lopes-Ferreira et al. on the predominance of this response profile in envenomation's caused by *Thallassophryne nattereri* (58).

In *Bothrops* snakebites, despite the small variety of molecules studied, it has been suggested that the circulating response is from the Th1 profile, but our data demonstrate that patients in the STC and MTC groups present a distinct response profile at the T0, T24, and T48 intervals (26, 49, 53). We believe that STC patients in T0 have a Th1 profile with a small participation of chemokines, which may cause an unsatisfactory response process for this condition, while MTC patients have a mixed profile with a large network of interaction between chemokines and inflammatory molecules (TNF and IL-6).

Due to the short period of recruitment time, the study had some limitations to the size of the sample and venom concentrations were not measured. In addition, the type of complication analyzed in this study is not too common in the field of snakebites, however it is one that has great clinical importance. And, even though we have not evaluated the use of adjunctive anti-inflammatory drug therapy, we describe possible therapeutic targets for future studies.

## Conclusion

In summary, the levels of circulating inflammatory soluble molecules are higher before antivenom administration in both groups, demonstrating an acute response to *Bothrops* snakebites. The molecules CCL-2 and CXCL-10, are shown as possible biomarkers for Severe Local Complication, although the CCL-2 stood out in all analyses. The circulating serum response profile differs between the studied groups, with the MTC patients presenting a mixed profile and STC patients presenting a more polarized profile for Th1 response in the T0. Besides which, individuals who develop STC have a more intense local immune response because the local response differs from the circulating response, which presents Th1/Th2/Th17 response polarization. Finally, we believe that the level of chemokines, anaphylatoxins and cytokines in circulation and the response profile they assume against *Bothrops* snakebite reflect the presence of the local complication and this can be used in clinical prognostic of the patients.

## Data Availability

All datasets generated for this study are included in the manuscript and/or the Supplementary Files.

## Ethics Statement

All protocols and consent forms were approved by the Research Ethics Committee at the FMT-HVD (CEP/FMT-HVD process #492.892/2014). Patients were treated according to the recommendations of Brazilian Health Ministry.

## Author Contributions

HI, AC, AM, and WM designed and performed the experiments, analyzed data, and wrote the manuscript. HI and AC analyzed data. HI, AC, JN, and MK performed the experiments. LF and MS revised the manuscript. HI, AC, WM, ML, AM, AT, JS, IS, and OM-F conceived and supervised the project, designed the experiments, interpreted the data, wrote, and revised the manuscript. All authors read and approved the final manuscript.

### Conflict of Interest Statement

The authors declare that the research was conducted in the absence of any commercial or financial relationships that could be construed as a potential conflict of interest.
