# Supplementary material for: An Immunological Stairway to Severe Tissue Complication Assembly in Bothrops atrox Snakebites
Source: Front Immunol. 2019 Aug 13;10:1882. doi: 10.3389/fimmu.2019.01882 (PMC6705225; doi:10.3389/fimmu.2019.01882)
Supplement: Supplementary file 1 [file Data_Sheet_1.docx]

**Supplementary Table 01:** Median and interquartile values (25% and 75%) of serum levels of immunological soluble molecules soluble molecules at admission (T0)

| Immunological soluble molecules | HD | MTC | STC |
| --- | --- | --- | --- |
| CXCL-8 (median [IQR]) | 391.1 [218.8-1191] | 525.5 [251.7-677.8] | 810 [287.4-1719] |
| CCL-2 (median [IQR]) | 821.5 [431.9-1887] | 1153 [193.1-1856] | 2099 [461.9-4579] |
| CXCL-9 (median [IQR]) | 1239 [277.4-1620] | 4728 [543.8-7909] | 5564 [1110-8516] |
| CCL-5 (median [IQR]) | 262143 [175691-262143] | 237028 [103488-262143] | 262143 [153233-262143] |
| CXCL-10 (median [IQR]) | 1228 [158.7-2279] | 2945 [194.5-3921] | 7896 [363.2-10687] |
| C3a (median [IQR]) | 1261 [622.1-1439] | 1199 [479.1-1457] | 840.0 [406.1]-1190] |
| C4a (median [IQR]) | 5010 [3622-5571] | 6011 [2418-7608] | 4377 [2091-5361] |
| C5a (median [IQR]) | 37332 [24150-47819] | 23202 [6052-27599] | 15568 [8753-21859] |
| IL-6 (median [IQR]) | 193.1 [105.8-166.1] | 233.1 [111.5-383.2] | 620 [164.5-1170] |
| TNF (median [IQR]) | 105.5 [84.37-120.1] | 95.8 [82.9-100.1] | 94.1 [85.1-104.0] |
| IL-1b (median [IQR]) | 0.02 [0.0-0.75] | 0.025 [0.0-0.11] | 0.05 [0.0-0.88] |
| IL-10 (median [IQR]) | 108.0 [ 94.38-120.1] | 154.4 [114.4-197.4] | 144.6 [123.0-419.5] |
| IL-2 (median [IQR]) | 153.0 [140.1-190.7] | 155.2 [145.9-161.6] | 162.8 [144.5-171.0] |
| IFN-y (median [IQR]) | 94.48 [82.94-105.1] | 93.66 [84.37-100.1] | 94.28 [79.38-00.74] |
| IL-4 (median [IQR]) | 165.4 [140.1-180.1] | 164.5 [150.2-173.0] | 165.1 [ 147.1-173.0] |
| IL-5 (median [IQR]) | 0.07 [0.0-0.11] | 0.4 [0.0-0.09] | 0.01 [0.0-0.12] |
| IL-17A (median [IQR]) | 103.0 [85.80-112.1] | 108.7 [80.08-137.6] | 111.6 [70.56-148.7] |
